# Strengthening and sustaining gender-based violence (GBV) coordination in emergencies: a synthesis of practitioner-driven, globally applicable recommendations

**DOI:** 10.1186/s13031-026-00770-9

**Published:** 2026-03-07

**Authors:** Philomena Raftery, Alina Potts, Melanie Hyde, Yermeyn York Wuyke, Hira Hashmey, Liliane Munezero, Ligia Kiss, Mazeda Hossain, Jennifer Palmer

**Affiliations:** 1 Department of Global Health & Development and Health in Humanitarian Crises Centre, London School of Hygiene and Tropical Medicine, Keppel Street, London, UK; 2 The Global Women’s Institute at the George Washington University, Washington DC, USA; 3 Technical Officer, Gender Equality, Health Equity and Human Rights, WHO Regional Office for Europe, Copenhagen, Denmark; 4 GBV/PSEAH specialist, Gender based violence area of responsibility (GBV AoR), Venezuela, Caracas, Venezuela; 5 GBV Specialist, United Nations population fund (UNFPA), Abuja, Nigeria; 6 GBV Specialist, United Nations population fund (UNFPA), Yaoundé, Cameroon; 7 Institute for Global Health, University College London, London, UK; 8 School of Social Sciences and the Eastern Africa Centre, Nottingham Trent University, Nottingham, UK

**Keywords:** Gender-based violence, GBV coordination, Humanitarian emergencies, Public health emergencies

## Abstract

**Introduction:**

Gender-based violence (GBV) is a global public health and human rights crisis, requiring coordinated efforts to ensure effective prevention, risk mitigation, and response, particularly in emergencies. Despite policy commitments underlining the importance of addressing GBV in emergencies, funding remains insufficient, inconsistent, and poorly aligned with aid prioritisation in the changing humanitarian and global health financing landscape. This paper synthesizes evidence on GBV coordination in humanitarian and public health emergencies and presents global recommendations to inform policy and practice in the context of the ongoing Humanitarian Reset.

**Methods:**

Using a three-phase qualitative methodology—comprising evidence synthesis, case study analysis, and a global expert practitioner consultation—we developed a framework and present strategic recommendations to strengthen and sustain GBV coordination in emergencies.

**Results:**

Our findings identify seven strategic recommendations aimed at investing in, sustaining, and transforming GBV coordination efforts globally. Key investment priorities include expanding the GBV coordination workforce, including for risk mitigation, prioritizing and systematically addressing GBV within public health emergencies, and investing in information management systems and strategic research. To sustain GBV coordination, we recommend adapting funding models, diversifying financial sources, advancing national leadership and localization, and implementing context-specific coordination approaches, including at the sub-national level. Furthermore, we propose that emergencies can serve as catalysts for broader social and legal transformations that advance GBV prevention and gender equality.

**Conclusion:**

Rather than accepting the deprioritisation of GBV coordination as an inevitable consequence of funding reductions framed as efficiency gains, our findings underscore the critical value of maintaining a dedicated focus on GBV within humanitarian coordination. Our findings provide practical, evidence-based recommendations and a global framework for policymakers, donors, and practitioners to strengthen and sustain GBV coordination in diverse emergency contexts. Sustained progress will require collective commitment to address GBV, even as funding landscapes change, and the backlash against gender equality continues to intensify.

## Key message

*What is already known on this topic*

Effective GBV coordination is critical in emergencies, but existing models face challenges due to limited evidence on functionality and effectiveness, fluctuating funding, and insufficient resourcing and investment. This study was needed to synthesize practitioner-driven insights and provide globally relevant, evidence-informed recommendations to strengthen GBV coordination in the context of a deepening humanitarian funding crisis, political pushback on women’s rights and gender equality, and failure to adequately prioritize GBV coordination as part of the Humanitarian Reset.

*What this study adds*

Drawing on a systematic synthesis of evidence, case studies, and expert practitioner consultations, this study presents seven globally applicable strategic recommendations. It also provides a comprehensive analytical framework for understanding the importance and value of specialised GBV coordination mechanisms in emergency settings. It examines how the integration of dedicated GBV coordination structures into broader protection coordination architectures, as envisaged under the Humanitarian Reset, may result in diminished access to essential, life-saving services for GBV survivors. Rather than accepting the deprioritisation of GBV coordination as an inevitable consequence of funding reductions framed as efficiency gains, the findings underscore the critical value of maintaining a dedicated focus on GBV within humanitarian coordination systems. Our recommended approach retains the accumulated expertise and practical experience of professionals working at the frontline of humanitarian emergencies and progress made over recent decades.

*How this study might affect research*,* practice or policy*

The findings offer actionable guidance for policymakers, donors, and practitioners to strengthen and sustain GBV coordination globally, particularly amidst evolving global health priorities and resource constraints. The recommendations can inform the development of flexible, context-sensitive coordination models, support investment in national capacities, and stimulate further research into the integration of GBV coordination across diverse emergency contexts. In a significantly altered humanitarian context where difficult prioritisation decisions are required, a focus on core, life-saving services must include the needs of GBV survivors as a non-negotiable component of the humanitarian response.

## Introduction

Gender-based violence (GBV) is a global public health crisis and a serious violation of human rights. Rooted in gender inequality, it disproportionately affects women and girls and results in profound public health, social, and economic consequences [1]. GBV includes sexual, physical, emotional, psychological, economic, institutional violence, and harmful traditional practices, all of which can result in serious health outcomes such as injury, psychological trauma, increased risk of sexually transmitted infections (STIs), including HIV, adverse reproductive outcomes, and mortality [2].

The increasing frequency, duration, and severity of humanitarian and public health emergencies amplify vulnerabilities to GBV while simultaneously disrupting health care and protective systems [1]. Globally, one in three women and girls experience physical or sexual intimate partner violence (IPV) or non-partner sexual violence during their lifetime [1–3]. Although data collection on GBV in emergencies remains challenging and likely underestimates true prevalence, existing evidence indicates elevated rates of sexual violence, IPV, technology-facilitated abuse, and reduced access to essential services [2, 4–6]. For example, in Ukraine, IPV reports to police increased by 40% in the first five months of 2023 compared to the same period in 2022, while conflict-related sexual violence continues to be documented [7]. Public health emergencies such as the Ebola and COVID-19 outbreaks have similarly intensified GBV risks while restricting service access during lockdowns [8].

Addressing the needs of GBV survivors requires comprehensive, coordinated response services delivered by diverse stakeholders at both global and field levels. GBV coordination has been a core component of humanitarian response architecture since 2005, intended to ensure that from the earliest stages of a crisis, survivors have access to safe, ethical, and survivor-centred services, while prevention and risk-mitigation measures are systematically implemented [9]. Within the United Nations (UN)–led cluster system, GBV coordination was situated under the Protection Cluster as a sub-sector, with global leadership provided through the GBV Area of Responsibility (AoR). Operationally, GBV coordination aims to convene UN agencies, national authorities, and international, national, and local actors to establish shared standards, clarify roles and responsibilities, identify and prioritise needs, address service gaps, and minimise duplication across actors [9]. Coordination mechanisms have until now been supported by tools such as the GBV Information Management System (GBVIMS), which facilitated the ethical collection, management, and use of data to inform collective decision-making and resource allocation [10]. Effective GBV coordination is also critical to enabling multi-sectoral action on risk mitigation, as articulated in the Inter-Agency Standing Committee (IASC) Guidelines for Integrating GBV Interventions in Humanitarian Action [11].

GBV coordination models vary by emergency context: in humanitarian conflict and disaster settings they are typically embedded in UN- and government-led cluster systems (with United Nations Population Fund (UNFPA) or United Nations High Commissioner for Refugees (UNHCR) in lead roles), whereas public health emergencies are organized through health-led incident management systems, usually under Ministries of Health with technical support from the World Health Organization (WHO), and rarely include formal GBV coordination mechanisms [12]. These differences highlight the need for adaptive, adequately resourced GBV coordination to ensure sustained prioritization and equitable access to services across emergency settings.

Despite progress driven by the UN cluster system (2005), the establishment of the GBV Area of Responsibility (AoR)(2006), and the global Call to Action on Protecting Women and Girls in Emergencies (2013), persistent challenges hinder effective GBV coordination in emergencies [1, 10]. Notwithstanding enhanced policy commitments and growing recognition of the importance of addressing GBV in emergencies, funding has remained insufficient, inconsistent, and poorly aligned with the changing humanitarian and global health financing landscape. Furthermore, a lack of published evidence on the effectiveness of GBV coordination has limited prioritisation, commitment and accountability across the humanitarian sector which has worsened under the Humanitarian Reset - a set of reforms called for by the UN’s Emergency Coordinator in response to the severe funding and legitimacy crises facing global aid in 2025 [13].

Compounding the situation, the recent global health and humanitarian funding cuts, particularly from the United States (U.S.) government and exacerbated by declining support from several European donors, has severely disrupted GBV coordination and responses in crisis-affected settings. Preliminary 2025 data from the GBV AoR indicated that the U.S. was the largest GBV donor in 2024, contributing USD 131.1 million, or 36% of the total GBV funding (USD 366.1 million) under the Global Humanitarian Overview [14]. Of 24 contexts with Humanitarian Response Plans, the US funded GBV programming in 19, amounting to USD 91.1 million. However, in 2025, US contributions markedly declined to USD 5.8 million, representing only 9.4% of the sector’s total USD 61.6 million [14]. This significant reduction has led to widespread service disruptions, weakened coordination mechanisms, and diminished national and local partner capacity [14]. International NGOs managing large GBV programs have faced substantial budget cuts, resulting in staff layoffs, furloughs, and program closures. Critical services, including women and girls’ safe spaces, cash and voucher assistance, and medical and psychosocial care for survivors of sexual violence, have been drastically curtailed, endangering survivors health and safety [14]. Similar reductions by other countries including traditional donors such as Germany, the UK, France and others have further narrowed the range of available funding options for the GBV response community.

In 2025, the Humanitarian Reset has signalled a substantive shift in the coordination of international humanitarian action, aiming to simplify structures, reduce fragmentation, and strengthen accountability to affected populations [13]. Over the past year, UN-led coordination is being reconfigured through this reform agenda, including the June 2025 IASC decision to consolidate the GBV, Child Protection, and Mine Action Areas of Responsibility within the Protection Cluster [13, 15]. Although intended to enhance coherence and efficiency, this consolidation is reshaping GBV coordination in emergencies by modifying established leadership, resourcing, and coordination arrangements. In several contexts, fears that GBV may lose visibility and technical prioritisation within broader protection structures, call for deliberate measures to preserve dedicated GBV expertise, uphold survivor-centred approaches, and ensure that GBV prevention and response remain integral to emergency operations rather than diluted within wider protection mandates, as highlighted to the Emergency relief coordinator by UNFPA and a consortium of women-led organizations in 2025 [16, 17]. During the 2025 Annual Partner Meeting of the Call to Action on Protection from GBV in Emergencies, participants highlighted the consequences of these dynamics for women and girls and for the sustainability of GBV services, with disproportionate impacts on local and women-led organisations [18]. While the Humanitarian Reset was widely perceived as posing risks to gender equality and gains made on addressing GBV, it was also framed as an opportunity to reaffirm GBV as a core humanitarian priority and strengthen leadership, accountability, financing, and support for locally-led GBV responses [18].

The original rationale for establishing GBV as a distinct coordination function should not be overlooked. In the absence of dedicated coordination mechanisms, GBV was largely rendered invisible within humanitarian responses, owing to limited documented cases, a lack of population-based prevalence data, and insufficient understanding among humanitarian actors of the ethical and safety constraints associated with GBV data collection. The establishment of specialised GBV coordination structures increased the visibility of GBV, promoted the principle of “action without evidence” by recognising GBV as an assumed and ongoing risk, enabled a dedicated humanitarian response, and strengthened GBV risk mitigation across sectors [9, 11]. Without this focus, GBV risks being marginalised within broader protection coordination. The Humanitarian Reset risks returning GBV work to its pre-2005 state, forcing the sector to relearn lessons and best practice already established over the past two decades.

Additionally, proposals to merge UNFPA and UN Women, framed as efficiency-driven reforms to reduce duplication, risk substantially weakening both agencies, as evidence indicates limited mandate overlap (20–30%) and highlights likely erosion of sexual and reproductive health and rights (SRHR) capacity, gender equality leadership, and specialized institutional infrastructure built over three decades [19]. Such consolidation represents not a neutral efficiency measure but a potentially harmful restructuring amid a global rollback on gender equality and SRHR.

This paper synthesizes recent evidence on GBV coordination in humanitarian and public health emergencies, and presents global recommendations to inform future policy and practice within this evolving context. We use new empirical evidence to refine a previously published framework for GBV coordination, drawing on a case study in Lebanon and a 2025 global consultation with GBV coordination experts [10]. We also examine the future of GBV coordination in light of shifting funding landscapes, including the withdrawal of U.S. and other government support and the Humanitarian Reset. The updated framework offers a globally relevant, practice-driven model for strengthening effective and context-sensitive GBV coordination.

## Methodology

This study employed a three-phase, qualitative approach to refine a globally relevant framework and synthesize key recommendations for strengthening and sustaining GBV coordination in emergencies. We integrated evidence synthesis, in-depth case study analysis, and a global expert practitioner consultation to ensure both rigour and relevance.

### Phase 1: Evidence synthesis

A 2022 scoping review synthesized global evidence from peer-reviewed and grey literature on GBV coordination in humanitarian and public health emergencies. The review identified 28 relevant publications which provided the foundation for an evidence-based framework highlighting key components needed for effective GBV coordination [10].

### Phase 2: Lebanon case study

Between 2019 and 2022, an in-depth qualitative case study was conducted to examine the evolution of GBV coordination in Lebanon’s protracted humanitarian crisis from 2012 to 2022. Lebanon’s context—marked by a large influx of Syrian refugees, restrictive refugee policies, prolonged political and economic instability, the COVID-19 pandemic, and subsequent compounded shocks including the Beirut Port explosion—offered critical insights into GBV coordination in complex and protracted emergencies. Findings from this work were previously published in two peer-reviewed papers [20, 21]. The study used a qualitative design informed by the previously published framework for effective GBV coordination. Data collection included 38 remote in-depth interviews with stakeholders involved in the GBV response, observations of GBV coordination meetings, and a review of key documents, conducted over a two-year period (October 2019–May 2022). A validation and sense-making workshop with GBV task force members and key informants in 2022 was used to refine and contextualise the analysis.

The case study documented how GBV coordination in Lebanon, initially established in response to the Syrian refugee crisis, evolved into a largely cohesive and effective national coordination mechanism. Findings highlighted the development of trust-based coordination structures, expansion of GBV services nationwide, and notable progress in localisation despite sectarian dynamics and a restrictive policy environment. The first publication illustrated how the protracted nature of the crisis acted as a transformative force for GBV coordination and service delivery, while also revealing significant limitations related to reliance on international leadership and funding, and weak government institutionalisation [21]. The second publication focused on Lebanon’s compounded crises from 2019 onwards, demonstrating adaptive and resilient coordination systems, alongside persistent challenges for local service delivery, engagement of refugee-led organisations, and long-term sustainability in the absence of stronger government leadership, relevant and inclusive conceptualisations of localisation and predictable, multi-year funding [20]. As this was the first dedicated study published on GBV coordination in a specific context, and given its depth and duration, the Lebanon case study informs a substantial proportion of the findings presented in this article, including challenges and subsequent recommendations.

### Phase 3: Global practitioner consultation

In February 2025, a global consultation was held with six GBV coordination experts with experience in Venezuela, Lebanon, Pakistan, Cameroon, Nigeria, South Sudan, Kenya, and the East African region. Through a semi-structured group discussion process, the experts reviewed findings from Phases 1 and 2 to reflect on their relevance to other settings, identify important themes in GBV coordination and reached consensus on key recommendations to enhance GBV coordination globally. Additionally, written input was integrated from a GBV specialist involved in the Ukraine crisis response, contributing further insights from an ongoing protracted humanitarian crisis. All practitioners involved in the consultation were offered the opportunity to collaborate as co-authors, reviewing the draft manuscript and strengthening the data analysis and recommendations.

### Ethics statement

Ethical approval for Phases 1 and 2 of the study was obtained from the London School of Hygiene and Tropical Medicine Observational / Interventions Research Ethics Committee (Project ID: 16208) in September 2019 and the institutional review board of the American University Beirut (Protocol Number: SBS-2020-0067) in February 2021. Phase 3 did not require ethics approval, as participants in the global consultation were considered collaborators and were included as co-authors; therefore, informed consent was not required.

## Results and discussion

**Fig. 1:**
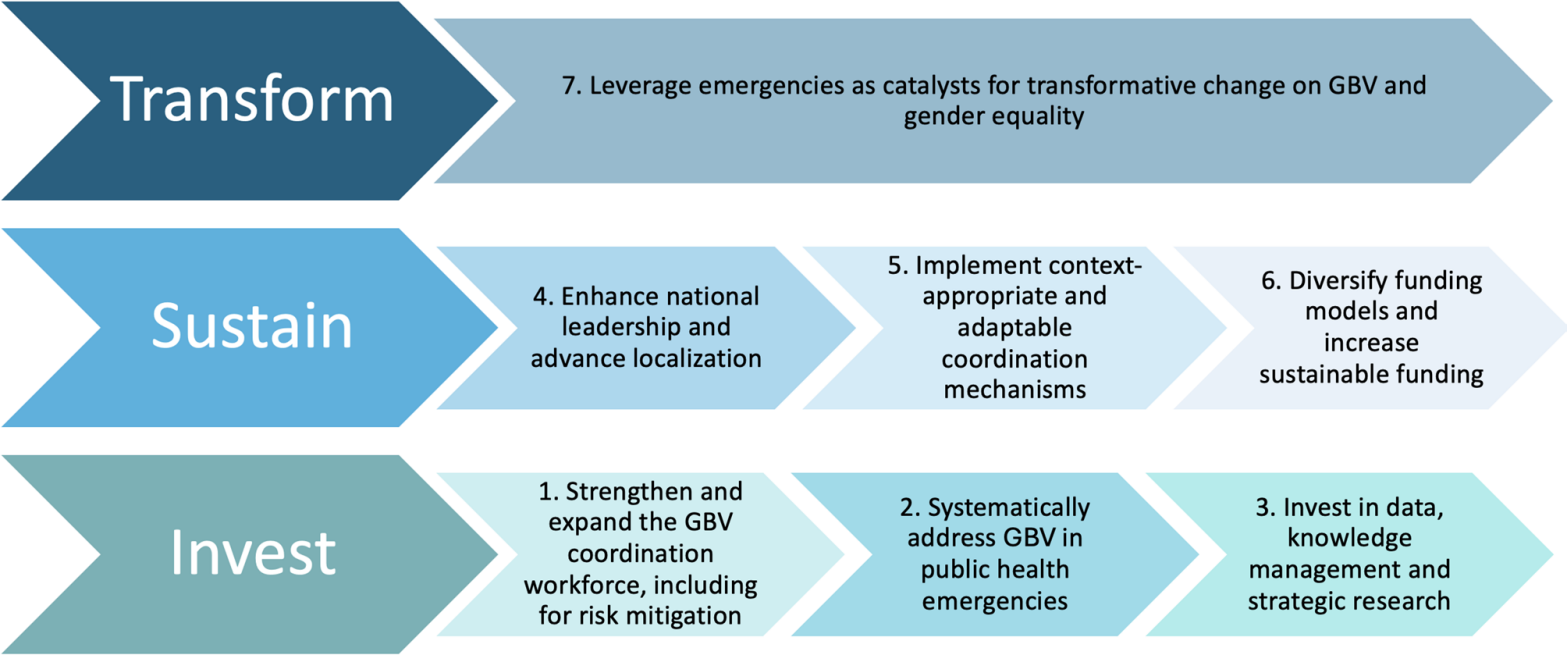
Global framework for strengthening and sustaining GBV coordination in emergencies

The results from the three phases of the study were synthesized into seven strategic challenges and recommendations, presented within a revised global framework for strengthening and sustaining GBV coordination (Fig. 1). Building on the original framework published in 2022, this updated version incorporates insights from the Lebanon case study and global consultation to address evolving challenges, emerging themes, and shifting priorities in GBV coordination [10]. The analysis generated seven key recommendations, organized across three interconnected dimensions — invest, sustain, and transform — to guide the sustaining and strengthening of GBV coordination in emergency contexts worldwide. Invest focuses on building the foundation for effective GBV coordination, including strengthening the workforce, prioritizing GBV in public health emergencies, and investing in data, knowledge management, and research. Sustain emphasizes the need to enhance national leadership, advance localization, implement adaptable coordination mechanisms, and diversify funding to ensure long-term stability. Transform highlights the potential to leverage emergencies as catalysts for systemic change in GBV and gender equality. These recommendations are further elaborated below, alongside the challenges they address.

### 1. Strengthen and expand the GBV coordination workforce, including for risk mitigation

#### Challenge

Skilled, experienced, and long-term dedicated GBV coordinators are critical for successful coordination, as they play a key role in fostering collaboration, mobilizing resources, and driving strategic decision-making [9]. Research underscores the influence of long-term GBV coordinators in shaping funding priorities, establishing data management systems, and sustaining coordination mechanisms [10, 21]. In Lebanon, leadership continuity was instrumental in building trust across national, local and international actors and strengthening sub-national and inter-agency collaboration [20, 21]. However, across multiple settings, high turnover rates, recruitment barriers, and having to manage dual operational and coordination roles continued to undermine the effectiveness of GBV responses [10]. Power imbalances and resource asymmetries for national organizations also limit their contributions to coordination [10]. The decline in US funding has jeopardized GBV coordination structures, with 40% of national coordinators and 35% of national co-coordinator positions across emergency settings dependent on US support [14]. In February 2025, key national coordination roles in Iraq, Venezuela, the Pacific, Mali, Afghanistan, Colombia, and Sudan faced possible termination, while sub-national coordination positions had already been eliminated in Sudan, South Sudan, Syria, and Ethiopia [14].

Although global guidance exists, GBV risk mitigation remains inconsistently integrated across sectors due to its frequent de-prioritization by non-GBV sectors and limited capacity of GBV coordinators to provide sustained support and capacity building amidst high humanitarian staff turnover [10, 22, 23]. Additionally, risk mitigation efforts in public health emergencies are frequently weak or absent, with responders often unaware of existing GBV guidelines [24]. While coordination teams offer technical guidance and advocate for integration—such as in Cameroon’s collaboration with WASH, nutrition, and food distribution sectors and Lebanons’ GBV risk mitigation mentorship approach —their influence on implementation is often limited because of power dynamics between sectors, donor-driven priorities and limited accountability mechanisms on GBV risk mitigation across sectors [21, 25].

#### Recommendation

Invest in building a skilled and experienced GBV coordination workforce across humanitarian and public health sectors. Early deployment of experienced coordinators—skilled in trust-building, collaboration, cultural competence, communication, and strategic planning—is essential in new crises. Capacity building of local staff and national and local women-led organizations should be adequately resourced to enable them to assume coordination roles. This is especially important in the context of the Humanitarian Reset and funding constraints as the cost of national positions is often a fraction of international deployments. As the humanitarian reform progresses and Humanitarian Coordinators are further empowered, strong advocacy with Humanitarian Coordinators in emergency settings regarding the value and importance of GBV coordination will become an increasingly critical priority for GBV experts.

Accountability for GBV risk mitigation must be reinforced by integrating GBV experts from the onset of emergencies, enforcing sector-wide standards, and mandating the inclusion and financial support of local women’s organizations in response planning [22, 26]. Donors should require explicit GBV risk reduction measures in all sectoral strategies [25]. Strengthening inter-cluster coordination is critical to address persistent gaps in role clarity and accountability that undermine effective GBV risk mitigation [27–29]. Close collaboration between Health Cluster and GBV actors is necessary to ensure clinical management of rape (CMR) and intimate partner violence (IPV) services are delivered as lifesaving interventions. Strategic models—such as Lebanon’s 2017 mentorship system and academic-civil society partnerships supporting coordination capacity transfer in countries neighboring Ukraine—offer promising approaches to enhance impact [6, 21].

### 2. Systematically address GBV in public health emergencies

#### Challenge

Despite mounting evidence that public health emergencies exacerbate GBV, it remains systematically deprioritized by public health institutions, governments, and donors as health systems become overwhelmed by the response. Previous and ongoing outbreaks—including Ebola, Zika, Mpox and COVID-19—have demonstrated how economic insecurity, social isolation, and disrupted health services, worsen structural inequalities and fuel GBV [8, 10, 30–32]. For instance, during the COVID-19 pandemic, resources were redirected towards outbreak control, away from essential GBV services, including through the repurposing of women-led safe spaces into isolation centers. Findings from the Global Health Cluster’s COVID-19 Task Team underscored the need for context-specific GBV risk assessments, improved referral pathways, and remote service provision to mitigate the barriers faced by survivors during public health crises [33].

Because public health emergency coordination models rarely include a dedicated or formal GBV coordination mechanism, GBV prevention and response must be systematically mainstreamed within existing health operations and emergency management functions. In recent years, WHO had strengthened its efforts to address GBV in health emergencies, including through the deployment of GBV technical capacity at global, regional, and country levels, particularly in graded humanitarian crises such as Gaza and Ukraine [34]. However, recent funding reductions threaten this capacity. In outbreak and epidemic responses, GBV continues to be under-resourced. WHO frameworks for health emergency preparedness and response provide limited explicit guidance on comprehensive GBV prevention, risk mitigation and response in public health emergencies [12, 35, 36].

#### Recommendation

Systematically integrate GBV response, risk mitigation and prevention into public health emergency preparedness and response policies and frameworks and health-led coordination. The inclusion of GBV expertise within WHO-led response teams is essential to ensure a robust health response and integration of GBV risk mitigation across all response functions. Investments in a trained GBV workforce dedicated to addressing GBV in public health crises is needed. In addition, mandatory GBV expertise and training should be required within Emergency Medical Teams (EMTs) deployed through WHO-coordination in emergency contexts [37]. Health emergency response coordination must be structured to enable responders to understand and address the GBV risks faced by women and girls in epidemics. Systematic attention to GBV in all aspects of epidemic preparedness and response is needed to ensure that future epidemic responses do not reinforce or deepen existing inequalities or cause unintended harm for women and girls and that GBV survivors can access life-saving services, including CMR and IPV services. Systematic roll-out and implementation of the Health and Protection Joint Operational Framework could support health actors to better meet the needs of GBV survivors [38].

Globally, GBV coordination and programming innovations piloted during COVID-19, including remote GBV service models, should be standardized and expanded for future emergencies [22, 24, 39]. In Lebanon, during COVID-19 the GBV task force successfully adapted referral systems, leveraged GBV data for coordination, and developed remote services for marginalized groups, including LGBTIQ+ individuals, migrants, persons with disabilities, and adolescent girls which could be replicated [20, 40, 41]. Women-led safe spaces must be preserved and prioritized as critical survivor-led platforms, and community engagement mechanisms should be leveraged for GBV prevention.

### 3. Invest in data, knowledge management and strategic research

#### Challenge

Effective GBV coordination relies on robust data and knowledge management systems, which require strategic investment in data collection, analysis, information management, research and communication [42]. Collecting and utilizing data on GBV is essential for planning context-appropriate interventions, yet this remains particularly challenging in emergencies due to the sensitive nature of the issue and operational constraints [43]. Common challenges include donor and agency leadership prioritizing data collection based on external reporting requirements rather than actionable insights, reluctance among organizations to share information, and the use of incompatible data collection tools across agencies, limiting comparability [28]. The implementation of the GBVIMS has significantly strengthened coordination in many settings; however, limited implementation, lack of dedicated information managers, and data sharing restrictions continue to impede strategic collaboration [42]. In Lebanon, GBVIMS was established as the first nationally coordinated GBV data system, providing standardized data that informed coordination and programming throughout both protracted and compounding crises [20, 21, 42, 44]. A dedicated GBVIMS coordination role was transitioned to a national staff member contributing to sustainability, and consistent data analysis informed coordination and programming [10, 20, 21]. In contrast, the absence of a dedicated GBVIMS information manager in Nigeria negatively affected data management efforts.

Furthermore, triangulating GBVIMS data with other sources is essential to develop a comprehensive understanding of GBV risks and support coordination [26]. Despite advancements in data collection tools, challenges remain in balancing standardization with contextual adaptation; for example, modifications to safety audit tools in Northeast Nigeria have compromised data quality and consistency. Strengthening interagency research and monitoring and evaluation (M&E) is also necessary to assess intervention effectiveness and guide implementation. Insufficient M&E staff presence in hard-to-reach areas of Syria and Nigeria have undermined service quality monitoring. A 2010 review of GBV research in Lebanon highlighted persistent challenges, including study duplication, fragmented funding, and the absence of a centralized repository, underscoring the need for a coordinated research platform [45].

#### Recommendation

Invest in dedicated information management positions and systems, such as GBViMS, that can harmonise and integrate GBV data and other sources and ensure field-level data is actionable for coordination and programming. Fully resource a strategic research agenda specificallly on GBV coordination at global, regional and country level, including in public health emergencies. Create a centralized repository for GBV research in emergencies to manage knowledge and learning, translate research evidence to policy and practice and promote cross-context learning to inform future responses. Strengthen data collection, analysis, and research strategies to enhance GBV coordination effectiveness, support evidence-based advocacy, and improve survivor-centered service delivery.

### 4. Enhance national leadership and advance localization

#### Challenge

While national governments hold the legal responsibility for coordinating humanitarian assistance, they are often marginalized within international coordination structures (28, 46). This reflects foreign actors’ perceptions that government counterparts lack capacity, may divert aid, or are unwilling to recognize GBV as a form of direct or indirect violence against civilians during conflict or displacement [46]. Some crisis-affected governments view GBV programming as foreign or imperialist, further complicating engagement [28, 47]. Lebanon’s experience illustrates both the potential and limitations of government-led coordination. While active leadership between 2015 and 2019, supported by a UN-funded position, helped stabilize the refugee responses through multi-year planning and humanitarian collaboration, fluctuating political will, limited institutional capacity, overwhelmed public institutions, and economic instability undermined the sustainability of GBV services and coordination [21, 48–50]. Similar challenges exist globally, where GBV remains insufficiently integrated into national policies and budgets. Sustained advocacy with economic and fiscal government actors is critical to demonstrate the long-term value of investing in GBV prevention and response.

National and local organizations, particularly civil society organizations (CSOs), often possess deep contextual knowledge and a nuanced understanding of community needs, positioning them to deliver culturally appropriate and effective interventions. However, national and local actors face significant barriers to engagement in humanitarian coordination dominated by international actors, including language difficulties, financial and logistical constraints, and limited familiarity with the system [28]. Moreover, discussing sensitive GBV-related issues in broader protection meetings may discourage the active participation of national and local GBV women’s organisations, particularly where women’s sexual and reproductive health rights are under threat, thereby undermining localisation objectives which the Humanitarian Reset explicitly seeks to strengthen [13]. In conflict or sectarian settings such as Lebanon, international actors may perceive local NGOs as lacking neutrality, while local organizations may feel marginalized by coordination mechanisms that prioritize international standards over local knowledge. Lebanon’s experience demonstrates how meaningful local engagement can strengthen GBV coordination. National and local CSOs played a critical role in sustaining and expanding services alongside government bodies, UN agencies, and international NGOs throughout the COVID-19 pandemic, the Beirut Blast, and in insecure areas [10, 20, 49–51]. However, further inclusion of Syrian refugee-led organizations in coordination, policy, and decision-making processes was needed [52, 53]. In contrast, the Haiti earthquake response illustrates the consequences of excluding local actors, as international agencies dominated coordination while disregarding local knowledge [10]. In Cameroon, the absence of a formally activated GBV sub-cluster led to reliance on regional sub-working groups, where local leadership eventually emerged following targeted UN-led capacity-building efforts. Persistent financial and logistical barriers require innovative and cost-effective approaches to strengthen local engagement, such as mobile training teams and regional knowledge-sharing networks. Recent findings from the GBV AoR’s February 2025 analysis on the impact of U.S. funding cuts highlight the vulnerability of national NGOs, which experienced the highest rate of funding suspensions (95%) and received the fewest waivers (5%) compared to international NGOs and UN agencies [14].

#### Recommendation

Enhance national GBV leadership and support strategic transitions from humanitarian-led to government-owned GBV coordination systems during protracted crises and recovery phases by integrating GBV responses into national health and social protection frameworks and promoting co-funding mechanisms. Marginalizing or excluding government actors risks undermining shared responsibility, trust-building, and long-term impact. Deliberate transition planning is essential to ensure the continuity of GBV coordination and services once emergency funding diminishes and international actors withdraw. Promising examples include government-led GBV sub-sectors in northeast Nigeria and the integration of GBV services into Pakistan’s national health budget. However, many governments lack the financial resources and technical capacity to sustain these efforts independently. High-level advocacy, including by Humanitarian and Resident Coordinators, as well as civil society-led advocacy within national parliaments and public forums, is critical to secure government co-funding and the integration of GBV services into national systems [46, 54]. The institutionalization of core GBV services, such as women’s safe spaces and case management—as implemented through Lebanon’s social development centres—can enhance sustainability beyond the crisis phase [21]. Capacity-building must also target government agencies and coordination bodies, as demonstrated in Cameroon and Lebanon, where government engagement enhanced GBV coordination and promoted the sustainability of interventions [21].

Promote localization to ensure sustained GBV coordination across all phases of acute emergencies, protracted crises, and recovery efforts [10, 22, 28, 48, 51]. This requires moving beyond tokenistic inclusion toward equitable leadership by local and women-led organizations in GBV coordination in line with the Humanitarian Reset objectives [18]. Long-term capacity-building, direct funding, and sustained partnerships are essential, aligning with global commitments to decolonize aid and redistribute power [21, 51]. Lebanon’s experience underscores the value of meaningful local engagement, where mutually beneficial partnerships between international and UN actors, national authorities, and CSOs enhanced GBV coordination throughout multiple crises [21]. Efforts to reinforce national coordination mechanisms—particularly those led by feminist organizations—are gaining traction in countries like Nigeria, where CSOs are taking on co-leadership roles at the state level. It is imperative that such gains are not lost through the Humanitarian reset. GBV case management task forces can serve as effective platforms for both coordination and capacity building, as seen in South Sudan and Lebanon [21]. During the COVID-19 pandemic, well-resourced and politically connected CSOs, alongside the presence of senior gender-focused government officials, played a pivotal role in advancing national GBV policies across multiple settings [54]. Global initiatives such as the Call to Action and the GBV AoR’s Global Localization Team should be expanded to further promote women’s leadership in GBV response [1, 22, 51, 55, 56].

### 5. Implement context-appropriate and adaptable coordination mechanisms

#### Challenge

Political, social, and cultural contexts can have a significant influence on GBV coordination and contextual understanding is critical to design and implement appropriate coordination mechanisms. In Lebanon’s sectarian political system, for example, coordinating multiple stakeholders with conflicting affiliations and political agendas posed challenges [21]. Patriarchal norms, weak legal protections, and overlapping crises heightened GBV risks, while restrictive refugee policies, low legal residency among refugees, and limited livelihood opportunities hindered survivors’ access to services and complicated their delivery, requiring careful navigation by GBV coordinators [21, 57]. Cultural sensitivities and limited government expertise also posed challenges for policy-level engagement on GBV [20, 21]. Understanding how policy actors frame GBV is essential to enable constructive dialogue; for instance, in Pakistan, modifying terminology to avoid sensitivities surrounding “gender-based violence” facilitated coordination efforts.

GBV coordination mechanisms must also respond to the evolving nature of crises. Failures in coordination during Typhoon Haiyan in the Philippines and the Syria crisis resulted in fragmented, reactive, and competitive responses [58]. Conversely, Lebanon’s coordination structures adapted over time to address overlapping mandates and crises [20, 28]. Evidence highlights the value of investing in subnational coordination, which enables faster, contextually informed decision-making and fosters the participation of operational actors, particularly CSOs [28, 59]. In Lebanon, subnational coordination with dedicated GBV coordinators played a key role in enhancing the participation of local CSOs and improving service delivery through a harmonized approach while addressing regional challenges, offering a replicable model for other contexts [21].

#### Recommendation

Establish context-specific and adaptable GBV coordination structures that are informed by a nuanced understanding of local sociopolitical and policy environments [20, 21]. Coordination mechanisms should be regularly reviewed and adjusted to reflect changing contexts, with particular investment in subnational coordination, localized guidance, and flexible operational approaches. National and local actors should be central to these coordinated, multi-sectoral efforts. Multisectoral GBV coordination is essential to link survivor-centred services with accountability and prevention by integrating health, legal, and judicial responses in ways that can deter future violence. Merging GBV coordination within protection structures may create opportunities to strengthen accountability and access to justice; however, without assured access to life-saving healthcare and essential MHPSS, survivors may face an increased risk of further harm if their immediate physical and mental health needs are not prioritised [17].

### 6. Diversify funding models and increase sustainable funding

#### Challenge

Despite heightened policy commitments to addressing GBV, existing funding mechanisms remain misaligned with the evolving humanitarian and global health financial landscape. Funding for GBV is both insufficient and inconsistent; over the past decade, only a minimal fraction of humanitarian aid has been allocated to this sector, including less than 1% of UN consolidated appeals in 2023 [22, 23]. Short-term funding cycles continue to hinder sustainable capacity building, limit prevention efforts, and restrict long-term systemic change [53]. In Lebanon, donor support and senior leadership helped prioritize and fund GBV; however, funding still only accounted for 1.3% of total humanitarian assistance in 2020, falling short amid escalating needs during compounded crises [20, 21]. Similarly, in protracted crises such as Cameroon and Northeast Nigeria, overall reductions in humanitarian aid have led to declining GBV resources despite rising needs and expectations to maintain innovation and impact. Beyond the amount of funding, the manner in which it is distributed poses challenges due to structural donor practices—such as imposing conditions, favouring large international NGOs over local organizations, and the politicization of funding.

Recent humanitarian funding cuts underscore the pressing need to diversify and stabilize funding streams. In countries like Cameroon and Venezuela, these cuts jeopardize nearly half of the GBV response budget, including funding for essential GBV coordination roles. Our Phase 3 study participants noted heightened competition for limited resources among both international and local NGOs, especially following the substantial decrease in U.S. GBV funding. Findings from the GBV AoR estimated that in Ethiopia, the suspension of U.S. funding forced 17 of 66 partners to stop operations, reducing GBV response capacity by 34% and cutting coverage from 38% to 24.5%, affecting nearly half a million people [14]. In Yemen, funding cuts are expected to impact 400,000 women and girls by closing 22 Safe Spaces, stopping cash assistance for > 9,700 survivors, and denying psychosocial support to 6,000 survivors [14]. Furthermore, GBV response actors must now navigate an increasingly complex advocacy landscape while seeking alternative financing mechanisms to maintain life-saving services. In response to U.S. funding cuts, advocacy strategies have shifted to align with changing political preferences, including the use of alternative terminology to preserve support for GBV efforts while resisting the erosion of feminist language.

#### Recommendation

Shift towards flexible, diversified, multi-year funding models that can adapt to respond effectively to rapidly evolving humanitarian contexts, moving beyond traditional donors [44, 52]. Strengthen advocacy efforts at both global and national levels to identify alternative financing mechanisms and secure sustainable, dedicated funding for GBV coordination and services, especially amid growing uncertainty in humanitarian and global health funding and reduced reliance on traditional donors [22]. Senior leadership within UN agencies, NGOs, and governments can play a pivotal role in securing adequate resources. Targeted awareness-raising—particularly aimed at Humanitarian Coordinators at the country level, who have increased decision-making authority over coordination structures and funding allocation under the Humanitarian Reset—can help sustain political will. Transitioning to needs-based funding allocation, coupled with innovative, coordinated advocacy and stronger government engagement, is imperative to ensure continuity of services in fragile and conflict-affected contexts. Promoting collaboration across organizations is equally important. Moving beyond siloed approaches, fostering collective thinking, and forming creative partnerships—such as consortiums between international and local NGOs—can help overcome financial barriers and strengthen technical capacity, as seen in Venezuela. Expanding global dialogue to share innovations and coordinate responses can further enhance collective impact. Strategies like those employed by the GBV task force in Lebanon to support local GBV staff through compounded crises could serve as adaptable models in other affected settings [20].

### 7. Leverage emergencies as catalysts for transformative change on GBV and gender equality

#### Challenge

Although emergencies exacerbate GBV risks, they can also serve as catalysts for transformative change when robust coordination mechanisms exist to sustain and scale such efforts. In Lebanon, the compounded crises prompted significant improvements in GBV coordination, evolving from weak initial structures into a comprehensive, multi-sectoral, survivor-centered framework [10, 21]. This progression laid the foundation for nationally led systems; however, as in other protracted crises, Lebanon’s GBV services remained heavily dependent on international funding, technical support, and leadership, particularly amid its economic crisis [20, 21]. Significant investments were made in expanding services, such as specialized legal and mental health support, as well as assistance for marginalized populations—including adolescent girls and LGBTIQ+ individuals—although these remained insufficient [20, 21, 50]. The compounded crises prompted coordinated GBV and mental health responses that integrated child protection, education, and psychosocial support to address issues like child marriage and adolescent girls’ needs [22, 57, 60–62]. Evidence from both Lebanon and South Sudan illustrates how coordinated, multisectoral approaches can embed GBV prevention and response within broader humanitarian and development systems, driving structural change.

More broadly, Lebanon’s protracted crisis served as a catalyst for significant legal and policy reforms over a decade, creating opportunities to reshape the landscape for women’s rights [21, 22]. Substantial progress was achieved in reforming GBV and gender-related policies, driven primarily by national and local actors, with humanitarian actors playing a catalytic role [21, 44, 50]. In Ukraine, the emergency response similarly prompted significant legal and policy changes that strengthened GBV prevention and response both domestically and in host countries receiving refugees [6]. In the WHO European Region, the designation of violence against women as a Special Initiative further illustrates how humanitarian crises can elevate GBV onto national and regional political agendas [63]. However, while these developments are promising, policy reforms and improved coordination alone are insufficient to achieve transformative change. Sustained progress requires confronting entrenched power dynamics and structural inequities within both humanitarian systems and affected societies at multiple levels.

#### Recommendation

Leverage emergencies strategically as opportunities to drive systemic transformation in GBV prevention and response. Ensure sustained, evidence-based investment in legal reforms, GBV prevention, and the institutionalization of coordination systems to achieve lasting, transformative change. Contextually tailored interventions that engage directly with underlying gender norms can yield sustained positive societal impacts [62]. Gender and protection actors should strengthen partnerships with feminist organizations, child protection actors, and other stakeholders to challenge harmful gender norms and shift power dynamics. In protracted crises, long-term prevention requires sustained efforts to address gender power dynamics across multiple levels of the socio-ecological framework [22]. Recovery planning should embed GBV prevention through ongoing investment in feminist movements, women’s leadership, economic empowerment, inclusive policy-making, and representative coordination structures. To advance GBV prevention in protracted emergencies, actors should adopt context-specific, multisectoral strategies grounded in robust evidence, including research from What Works, systematic reviews, and UN prevalence data [64]. Targeted adolescent programming offers an important opportunity to address specific GBV risks such as child marriage, trafficking, and female genital mutilation. Similarly, greater utilization of the Women, Peace and Security agenda can institutionalize gains, strengthen local organizations, and advance prevention efforts by embedding gender equality in peacebuilding and recovery processes.

## Conclusion

This paper examines current challenges in GBV coordination in humanitarian and public health emergencies, drawing on prior research, insights from GBV practitioners, and updated global evidence, while reflecting on the impact of the humanitarian reset and funding cuts across the sector. We present a refined globally-relevant framework for strengthening and sustaining GBV coordination in emergencies. Seven strategic recommendations were developed and consolidated, focusing on investment, sustainability, and transformation of GBV coordination globally. These foci include expanding the GBV coordination workforce, systematically addressing GBV within public health emergencies, and strengthening information management and strategic research capacities. The framework also highlights the multiple risks posed by the proposals to consoldiate GBV coordination within the protection sector. To sustain GBV coordination, we recommend adapting funding models, diversifying financial sources, advancing national leadership and localization, and implementing context-specific coordination approaches, including at the sub-national level. Finally, we highlight how emergencies offer opportunities for transformative change when power hierarchies are addressed and systemic inequities confronted in ways that reinforce longer-term efforts led by feminist organizations.

This paper is among the first to examine contemporary challenges in global GBV coordination systems. It triangulates findings and synthesizes evidence from a literature review, case study, and consultation with experts working across diverse crisis contexts. Its findings are, however, constrained by several factors: the scarcity of robust evidence on GBV coordination and responses across different geographies; the rapidly evolving humanitarian coordination landscape; and limited visibility of longer-term impacts. Nonetheless, the paper provides a novel perspective on the current state of GBV coordination and offers practical recommendations to sustain existing efforts and guide future investments.

The current global landscape, particularly the withdrawal of U.S. and European governments’ funding and the ongoing Humanitarian Reset, underscores the fragility of existing GBV coordination mechanisms and highlights the urgent need for sustained political and financial commitment. To ensure that GBV remains a core humanitarian priority, stakeholders at national, regional, and international levels must mobilize long-term, flexible resources, advocate for enabling policies including within the Humanitarian reset, and invest in and sustain GBV coordination systems. The future of GBV coordination depends on collective action not only to respond to GBV but also to prevent and mitigate it, particularlly amid the deepening humanitarian funding crisis, political backlash on women’s rights and gender equality, and fundamental changes to the coordination of a humanitarian system that has historically struggled to meet the needs of GBV survivors.

## Data Availability

The datasets analysed during the current study are available from the corresponding author on reasonable request.

## Declarations

### Disclaimer

Any opinions stated within this document reflect those of the authors and not necessarily of the United Nations Population Fund or the World Health Organization.
